# Course of depressive symptomatology and its association with serum uric acid in one-anastomosis gastric bypass patients

**DOI:** 10.1038/s41598-020-75407-9

**Published:** 2020-10-27

**Authors:** Eva Winzer, Bernhard Ludvik, Igor Grabovac, Renate Kruschitz, Karin Schindler, Gerhard Prager, Carmen Klammer, Friedrich Hoppichler, Rodrig Marculescu, Maria Wakolbinger

**Affiliations:** 1 Department of Social and Preventive Medicine, Centre for Public Health, Medical University of Vienna, Vienna, Austria; 2 Special Institute for Preventive Cardiology and Nutrition-SIPCAN, Salzburg, Austria; 3 Division of Endocrinology and Metabolism, Department of Internal Medicine III, Medical University of Vienna, Vienna, Austria; 4 Department of Medicine 1 and Karl Landsteiner Institute for Obesity and Metabolic Diseases, Rudolfstiftung Hospital, Vienna, Austria; 5 Division of Internal Medicine, General Public Hospital of the Order of Saint Elisabeth, Klagenfurt, Austria; 6 Division of General Surgery, Department of Surgery, Medical University of Vienna, Vienna, Austria; 7 Department of Internal Medicine, Convent of the Brothers of Saint John of God Linz, Linz, Austria; 8 Division of Internal Medicine, General Public Hospital of the Brothers of St. John of God Salzburg, Salzburg, Austria; 9 Clinical Institute for Medical and Chemical Laboratory Diagnostics, Department of Laboratory Medicine, Medical University of Vienna, Vienna, Austria

**Keywords:** Gastrointestinal system, Biomarkers, Risk factors

## Abstract

The changes in depressive symptomatology during the first year following one-anastomosis gastric bypass (OAGB) were evaluated and its association with uric acid (sUA). Fifty patients were included in this analysis. Beck Depression Inventory (BDI) for measuring depressive symptomatology, blood samples, and anthropometric measurements were assessed before (T0), at 6 (T6), and 12 months (T12) after surgery. There was a significant reduction in BDI total score at T6 (− 5.6 (95% CI − 2.1, − 9.1) points; p = 0.001) and at T12 (− 4.3 (95% CI − 0.9, − 7.9) points; p = 0.011). BMI loss was unrelated to depressive symptomatology. Patients with moderate to severe depressive symptomatology presented lower sUA levels than patients with none or minimal to mild (p = 0.028). ROC analysis revealed that sUA levels below 5.0 at T6 and 4.5 mg/dl at T12 had a prognostic accuracy for depression severity. Furthermore, delta sUA was significantly associated with delta BMI (β = 0.473; p = 0.012) and delta waist circumference (β = 0.531; p = 0.003). These findings support an improvement in depressive symptomatology in the first year postoperatively, however, without relation to BMI loss. Patients with moderate to severe depressive symptomatology presented with lower sUA levels over time. Therefore, sUA could be useful to predict moderate to severe depressive symptomatology in patients undergoing OAGB in clinical practice.

## Introduction

Presently, bariatric surgery is the most effective weight loss method in adults with morbid obesity, who have been unsuccessful with nonsurgical interventions^1,2^.

Obesity and major depressive disorder are important public health problems connected to high disease burden, health care expenses and present great challenges for the upcoming decades^3,4^. Depressive symptoms are highly prevalent co-morbidities occurring in patients with severe obesity^5^. Various studies have reported a correlation between obesity and depressive symptoms compared to controls^6–8^. Furthermore, detecting depression in candidates for bariatric surgery is important as the presence of depressive symptoms prior to surgery has been associated with poor outcomes and regaining the lost weight^9,10^. However, up to date, no study has investigated the course of depressive symptoms following one-anastomosis gastric bypass (OAGB). OAGB is the third most common bariatric procedure worldwide^11^.

Additionally, major depressive disorder has been found associated with oxidative stress pathways and altered purinergic metabolism^12^. This is of significance as increasing evidence shows the important role of the purinergic system in mood regulation, motor activity, cognitive function, sleep, and behaviour^13^ and its impairment seems to be implicated in the pathophysiological mechanisms of depressive symptoms^14^. As serum uric acid (sUA) is the end product of purine metabolism^15^, patients with major depressive disorder have been found to have lower levels of sUA compared to those without major depressive disorder^12^. Moreover, recent evidence suggests that sUA concentrations were strongly associated with body weight in adults^16^.

Utilizing data from the LOAD (“Link between Obesity And Vitamin D”) study, a six-months double-blind, placebo-controlled, randomized trial of vitamin D supplementation in OAGB patients, the aims of the current analysis are: (1) to describe changes in depressive symptomatology during the first year following surgery; (2) to examine whether depressive symptomatology changes in parallel with total body weight loss; (3) to determine the association between depressive symptomatology and sUA and (4) to examine whether decreases in sUA levels would correlate with weight change.

## Materials and methods

### Study design

This paper presents a secondary analysis of the data from a six-months double-blind, placebo-controlled, randomized trial of vitamin D supplementation: “Link between Obesity And Vitamin D”^17^. The study protocol was previously published^18^. In the first six months after OAGB surgery, the intervention group received the following vitamin D dosing regimen: Up to three oral loading doses of each 100,000 IU in the first month postoperatively followed by maintenance dose of 3420 IU/day; and the control group received conventional supplementation (placebo with following maintenance doses of 3420 IU/day). Afterwards, both groups were recommended to continue the vitamin D_3_ supplementation until the follow-up visit at 12 months. At baseline, no participant took any vitamin D supplementation, which was an inclusion criterion for this trial. Inclusion criteria were; planned OAGB surgery, above 18 years old, 25-hydroxy-vitamin D (25(OH)D) serum concentrations of < 75 nmol/L, and a body weight < 140 kg (due to body weight limitation of the dual energy X-ray absorptiometry). Specific exclusion criteria included any other planned form of bariatric surgery than OAGB, hypo- and hypercalcemia, renal insufficiency, or primary hyperparathyroidism. The details on design, the used materials and methods, as well as the sample size calculation of the study have been previously published^18^.

All OAGB procedures were performed at the General Hospital Vienna, Medical University of Vienna by the same surgical team using a laparoscopic approach. It is a simplified procedure that consists of a unique gastrojejunal anastomosis between a 30 to 40 ml sleeve gastric pouch and a jejunal omega-loop of approximately 200 cm^19^. The study methods are in accordance with the CONSORT (Consolidated Standards Of Reporting Trials) guidelines for reporting randomized trials^20^.

This study was approved by the local Ethics Committee of the Medical University of Vienna (No° 1899/2013), by the Austrian Competent Authority (No° LCM-718280-0001), registered at clinicaltrials.gov (Identifier: NCT02092376) and EudraCT (Identifier: 2013-003546-16), complies with the Declaration of Helsinki^21^, and conducted from April 2014 to June 2016 at the Medical University of Vienna (Austria). The study participants were scheduled for OAGB surgery and all of them gave written informed consent preoperatively.

### Assessment of variables

Data were assessed before surgery (T0), at 6 months (T6), and at follow-up visit at 12 months postoperatively (T12). At T0, age, sex, medical history (e.g. comorbidities, prescribed medication) were collected as previously described^18^. Clinically diagnosed depression, was defined whether the patients were ever diagnosed with depression by a health professional e.g. psychologist or had taken antidepressant medications. The following set of evaluations was obtained for each participant at the three time points: height and body weight (measured with the calibrated scale Seca mBCA 515) and waist circumference measured with an inelastic tape at the narrowest point between the lower costal border and the top of the iliac crest in accordance with the International Standards for Anthropometric Assessment (ISAK)^22^.

Blood samples were collected and serum uric acid (sUA), using uricase-PAP-method, was used for this analysis.

### Beck Depression Inventory

The Beck Depression Inventory (BDI)^23^ is a 21-question multiple-choice self-report inventory for measuring the severity of depressive symptomatology. Symptoms of depression over the past one week were assessed using the BDI version 1^23,24^. Because many patients are advised to lose weight in preparation for surgery, no points were assigned to the BDI item, “I have lost more than 5 lb,” for participants who indicated purposefully trying to lose weight. The following cut-offs were used for the total score: 0–9, 10–18, 19–29 and 30–63, reflecting none or minimal, mild, moderate and severe symptomatology, respectively^25^.

Somatic and physical items of the BDI, such as distortion of body image, work inhibition, sleep disturbance, fatigability, loss of appetite, somatic preoccupation, loss of libido, and weight loss can overlap with the symptoms of obesity rather than being due to depressive symptomatology^26,27^. Previously published studies reported that patients with obesity or patients undergoing bariatric surgery endorsed the somatic-vegetative items more than the cognitive affective items of the BDI^28,29^. The initial validation study by Steer et al. with adult psychiatric outpatients using both exploratory and confirmatory factor analyses found two-factor solutions including somatic-affective and cognitive factors^31^. Therefore, the items of the BDI have been divided into two subscales: the cognitive-affective subscale which assesses the mental aspect of depression (BDI items 1–13) and the somatic-vegetative subscale which measures vegetative and somatic symptoms (BDI items 14–21). This was also used in the study by Jorde et al.^30^.

### Statistical analysis

The results are expressed as mean (standard deviation) for continuous and as percentages for categorical variables. In order to test for normal distribution, a visual test (histograms and box plots) was used and the Kolmogorov–Smirnov test was applied in addition.

The internal consistency of the BDI items was determined by a reliability analysis (Cronbach’s alpha) and showed a Cronbach’s alpha of 0.895, which shows that the questionnaire is reliable in this population.

The main outcome of interest was the change in BDI scores in the first postoperative year. In this secondary analysis, there was no significant effect of vitamin D supplementation (intervention or control group) nor of vitamin D status (25(OH)D) on Beck Depression Inventory (BDI) score. Therefore, the entire sample for this analysis was used. Repeated-measures analysis of covariance was applied to assess the effect of time, by using different covariance structure models as appropriate and were adjusted for age, sex, season, and baseline values to supply an unbiased estimate of the mean difference^31^. A post-hoc analysis with Bonferroni correction was used. Additionally, linear mixed models were performed to assess associations between changes in body weight and BMI and the depressive symptomatology.

Estimates of the prevalence of depressive symptomatology over time were calculated using generalized estimating equation (GEE) with a logit link function for binary outcomes and unstructured covariance matrices. With this approach, effects with time as repeated factor with depressive symptomatology as dependent variable was examined, adjusted for age, sex, season, and baseline values.

Moreover, to examine the effects of time (from baseline to 6 and 12 months), group (depressive symptomatology), and their interaction on sUA, GEEs were used. Linear link function and an unstructured correlation matrix were used. Significance tests were performed with Wald χ^2^ (α = 0.05). The models were adjusted for age, sex, and season. Baseline data and none or minimal to mild depressive symptomatology were the reference. For detection the optimal cut-off in sUA levels for predicting moderate to severe depressive symptomatology at different time points, receiver operator characteristic curves (ROC) were used and described as area under the curve (AUC) with standard errors, the negative (NPV) and positive predictive value (PPV).

The statistical association between sUA concentrations and BDI scores with anthropometric parameters such as weight, BMI and waist circumference was examined by multiple linear regression analysis.

Means were compared unadjusted without imputation of missing data. All statistical analyses were performed with IBM SPSS Statistics for Windows, Version 23 software (IBM Corporation, Armonk, New York, USA). P-values < 0.05 were considered statistically significant and all tests were two-sided.

### Statements regarding ethics and consent

All procedures performed in studies involving human participants were in accordance with the ethical standards of the institutional and/or national research committee and with the 1964 Helsinki declaration and its later amendments or comparable ethical standards. Informed consent was obtained from all individual participants included in the study.

## Results

### Patients’ characteristics

Out of 67 eligible patients, 17 declined to participate (25%). The remaining 50 patients entered the randomized controlled trial^17^ with 39 patients who completed the BDI questionnaire at baseline. Baseline characteristics are demonstrated in Table 1. Before surgery, 23% of the patients had a clinically diagnosed depression, 20% among them were using antidepressant medications. However, 56% of the patients showed moderate to severe depressive symptomatology as measured by the BDI.

**Table 1:** Initial patients' characteristics and comorbidities.

	Total (n = 39)
Female, n (%)	32 (82)
Age (years)	42.5 (12.5)
Weight (kg)	120.9 (13.4)
BMI (kg/m^2^)	44.2 (4.1)
Waist circumference (cm)	129.3 (10.0)
**Comorbidities (anamnesis)**	
Diabetes mellitus, n (%)	13 (33)
Hypertension, n (%)	21 (53)
Dyslipidemia, n (%)	13 (33)
Depression, n (%)	9 (23)
Antidepressant drug use, n (%)	8 (20)
Smokers, n (%)	7 (18)
**BDI total score**	21.1 (9.6)
None or minimal symptomatology, n (%)	4 (10)
Mild symptomatology, n (%)	13 (33)
Moderate symptomatology, n (%)	12 (30)
Severe symptomatology, n (%)	10 (25)
**BDI subscales**	
Cognitive-affective	11.9 (6.3)
Somatic-vegetative	9.2 (4.1)

Data are presented as mean (standard deviation) or percentages.

*BMI *body mass index, *BDI* Beck Depression Inventory.

### Changes in depressive symptomatology in the first postoperative year

Table 2 shows the change in weight and depressive symptomatology over time. As expected, there was a significant BMI change (p < 0.001) and total body weight loss (p < 0.001) over time. There was also a significant reduction in BDI total score between baseline and 6 months (21.1 ± 9.6 vs. 14.0 ± 7.7 points; p = 0.001), and baseline and 12 months (21.1 ± 9.6 vs. 16.0 ± 11.3 points; p = 0.011). The analysis at 6 and 12 months showed a significant improvement of the score in the subscale somatic-vegetative (T6 p < 0.001, T12 p = 0.003), as well as for the items “fatigability” (T6 p = 0.001, T12 p = 0.007), “loss of appetite” (T6 p = 0.018, T12 p = 0.015), and “somatic preoccupation” (T6 p < 0.001, T12 p < 0.001). The cognitive-affective subscale analysis had also a significant improvement at 6 months (p = 0.001) but the change between baseline and 12 months was not significant (p = 0.283). The items of the cognitive-affective subscale showed a significant improvement in “guilty feeling” (T6 p = 0.014), “sense of punishment” (T12 p = 0.048), “indecisiveness” (T6 p = 0.002, T12 p = 0.021), and “distortion of body image” (T6 p = 0.016, T12 p = 0.030). The complete analysis with the subscales can be observed in Table 3.

**Table 2:** Change in weight and depressive symptomatology over time.

	Baseline (T0)	6 months (T6)		p-values+	12 months (T12)		p-values+	p-values (time)+
	Mean (SD)	Mean (SD)	Difference*		Mean (SD)	Difference*		
			Mean (95% CI)			Mean (95% CI)		
Weight (kg)	120.9 (13.4)	87.8 (11.1)	− 32.3 (− 29.2, − 35.3)	*< 0.001*	79 (11.3)	− 42 (− 38.8, − 45.2)	*< 0.001*	*< 0.001*
BMI (kg/m^2^)	44.2 (4.1)	32.3 (4.0)	− 11.8 (− 10.7, − 12.8)	*< 0.001*	28 (3.8)	− 15.3 (− 14.1, − 16.4)	*< 0.001*	*< 0.001*
Waist circumference (cm)	129.3 (10.0)	102.8 (9.2)	− 26.0 (− 22.2, − 29.8)	*< 0.001*	97.4 (10.2)	− 32.1 (− 28.1, − 36.1)	*< 0.001*	*< 0.001*
BDI total score	21.1 (9.6)	14.0 (7.7)	− 5.6 (− 2.1, − 9.1)	*0.001*	16.0 (11.3)	− 4.3 (− 0.9, − 7.9)	*0.011*	*< 0.001*
Subscale cognitive-affective	11.9 (6.3)	7.4 (4.4)	− 3.3 (− 1.2, − 5.4)	*0.001*	9.0 (7.2)	− 1.9 (0.8, − 4.6)	*0.283*	*0.001*
Subscale somatic-vegetative	9.2 (4.1)	6.5 (3.9)	− 2.3 (− 1, − 3.6)	*< 0.001*	6.0 (4.8)	− 2.4 (− 0.7, − 4.0)	*0.003*	*< 0.001*

Data are presented as mean (standard deviation) and the difference between time as mean (95% confidence interval); ^+^Repeated measure analysis of variance and post hoc analysis with Bonferroni correction, adjusted for baseline value, age, sex, (and season).

At baseline: n = 39, at 6 months: n = 36, and at 12 months: n = 33.

Significant findings are in italics.

*BMI* body mass index, *BDI* Beck Depression Inventory.

**Table 3:** Beck Depression Inventory questions during the first year postoperatively.

	Baseline (T0)	6 months (T6)	p-values^+^	12 months (T12)	p-values^+^	p-values (time)^+^
	Mean (SD)	Mean (SD)		Mean (SD)		
**Subscale cognitive-affective**						
Sadness	1.2 (0.5)	0.9 (0.7)	0.443	1.0 (0.7)	0.201	0.130
Pessimism	0.9 (0.8)	0.7 (0.9)	1.000	0.8 (0.9)	1.000	0.729
Sense of failure	0.8 (0.8)	0.4 (0.5)	0.299	0.6 (0.8)	1.000	0.256
Lack of satisfaction	1.0 (0.8)	0.6 (0.6)	0.079	0.8 (0.7)	0.619	0.074
Guilty feeling	0.9 (0.9)	0.3 (0.5)	*0.014*	0.8 (1.0)	1.000	*0.014*
Sense of Punishment	0.8 (0.9)	0.4 (0.5)	0.070	0.3 (0.6)	*0.048*	*0.019*
Self-dislike	1.0 (0.7)	0.6 (0.5)	0.134	0.6 (0.8)	0.274	0.078
Self-criticalness	1.1 (0.8)	0.6 (0.6)	0.095	1.0 (0.8)	1.000	0.097
Suicidal wishes	0.1 (0.3)	0.0 (0.0)	1.000	0.2 (0.4)	0.511	0.236
Crying	0.9 (0.7)	0.6 (0.7)	0.051	0.7 (0.9)	0.517	*0.050*
Irritability	1.2 (0.7)	1.0 (0.7)	0.113	1.2 (0.9)	1.000	0.113
Social withdrawal	0.8 (0.9)	0.6 (0.6)	0.302	0.6 (0.9)	0.851	0.226
Indecisiveness	1.2 (0.7)	0.7 (0.5)	*0.002*	0.8 (0.6)	*0.021*	*0.001*
**Subscale somatic-vegetative**						
Distortion of body image	1.6 (1.1)	1.0 (0.8)	*0.016*	1.2 (0.9)	*0.030*	*0.005*
Loss of libido	0.9 (0.9)	0.7 (0.9)	0.714	1.0 (1.1)	1.000	0.412
Work inhibition	1.4 (1.1)	0.8 (0.7)	0.102	1.0 (0.9)	0.146	*0.046*
Sleep disturbance	1.1 (1.2)	1.0 (1.1)	0.606	1.1 (1.1)	0.332	0.210
Fatigability	1.5 (0.9)	1.0 (0.8)	*0.001*	1.0 (1.0)	*0.007*	< *0.001*
Loss of appetite	0.6 (0.6)	1.0 (0.8)	*0.018*	1.0 (0.7)	*0.015*	*0.004*
Somatic preoccupation	2.1 (1.0)	1.1 (0.6)	< *0.001*	1.0 (0.9)	< *0.001*	< *0.001*

Data are presented as mean (standard deviation); ^+^Repeated measure analysis of variance and post hoc analysis with Bonferroni correction, adjusted for baseline value, age, sex, and season.

At baseline: n = 39, at 6 months: n = 36, and at 12 months: n = 33.

Significant findings are in italics.

**Figure 1:**
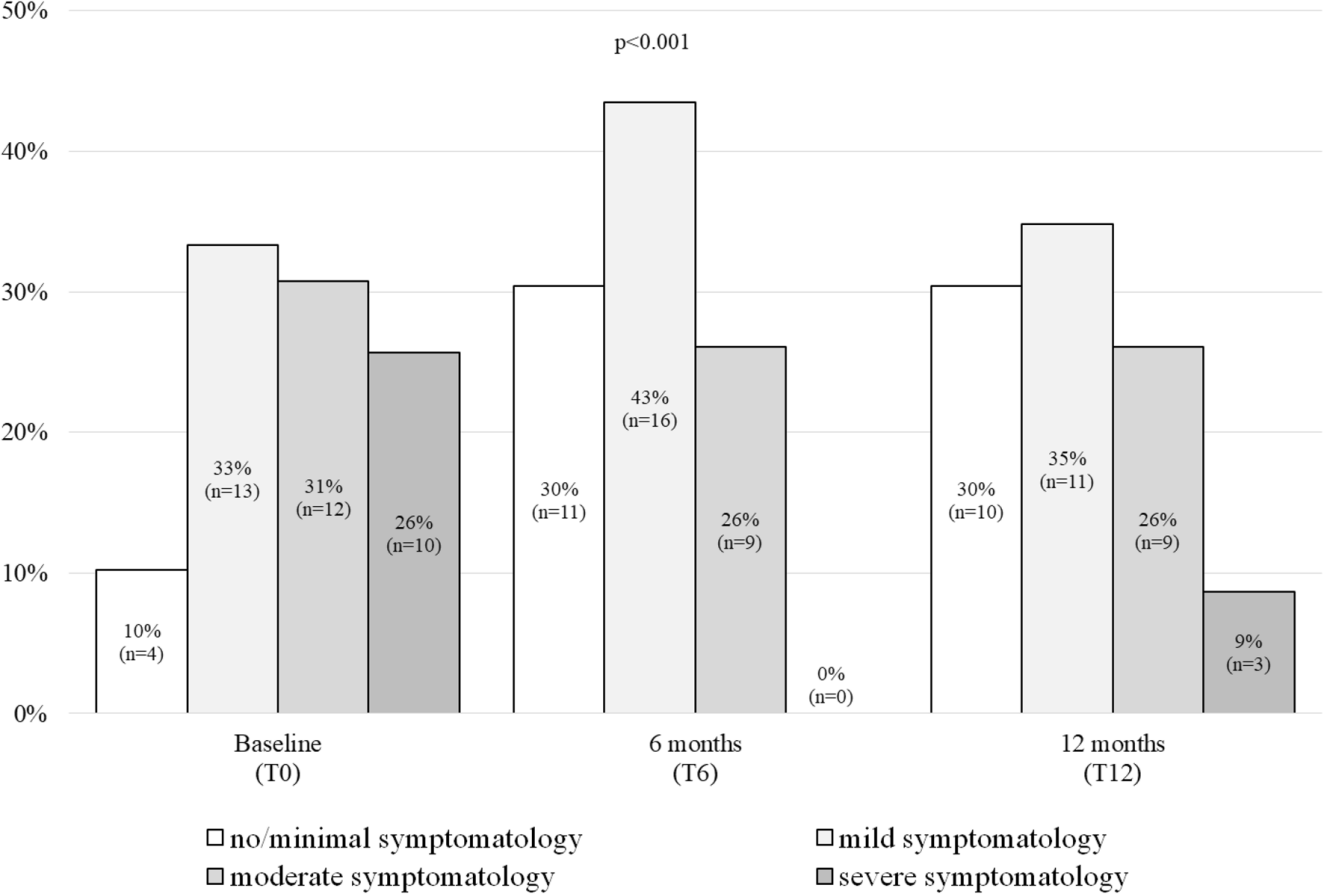
Change in depressive symptomatology over time. P values were based on Generalized Estimating Equations to examine the effects of time (from baseline to 6 and 12 months) with the prevalence of depressive symptomatology as the dependent variable. Logit link function and an unstructured correlation matrix were used. Significance tests were performed with Wald χ^2^ (α = 0.05). The models were adjusted for age, sex, and season. Baseline data was the reference.

Figure 1 shows the percentage change in depressive symptomatology over time. The prevalence of moderate to severe depressive symptomatology decreased from 56% at baseline to 26% at 6 months and increased slightly to 35% at 12 months. The prevalence of none or minimal to mild depressive symptomatology increased from 44% at baseline to 74% at 6 months and to 65% at 12 months (p = 0.045). The adjusted OR for moderate to severe depressive symptomatology at 6 months was 0.28 (95% CI 0.12, 0.62; p = 0.002), and at 12 months 0.37 (95% CI 0.14, 1.0; p = 0.051) compared to baseline.

### Depressive symptomatology and body weight loss

The baseline weight, BMI, and waist circumference parameters of patients with no or minimal to mild vs. moderate to severe depressive symptomatology were similar with 124.0 (17.0) kg vs. 120.5 (13.2) kg (p = 0.940), 44.9 (4.6) kg/m^2^ vs. 44.1 (4.1) kg/m^2^ (p = 0.800), and 124.5 (11.5) vs. 129.9 (9.8) cm (p = 0.086), respectively.

Furthermore, in the longitudinal course, patients with no or minimal to mild depressive symptomatology showed a 29.8 (6.6) % and 36.5 (9.5) % Total Body Weight Loss (TBWL) and patients with moderate to severe depressive symptomatology showed a 26.2 (5.4) % and 33.9 (6.3) % TBWL at 6 and 12 months (p = 0.367), respectively. Moreover, there were no significant associations between BMI loss and the change of BDI total score at 6 (β = 0.103; p = 0.641) and 12 months (β = 0.032; p = 0.886) after surgery.

### Depressive symptomatology and sUA

**Figure 2:**
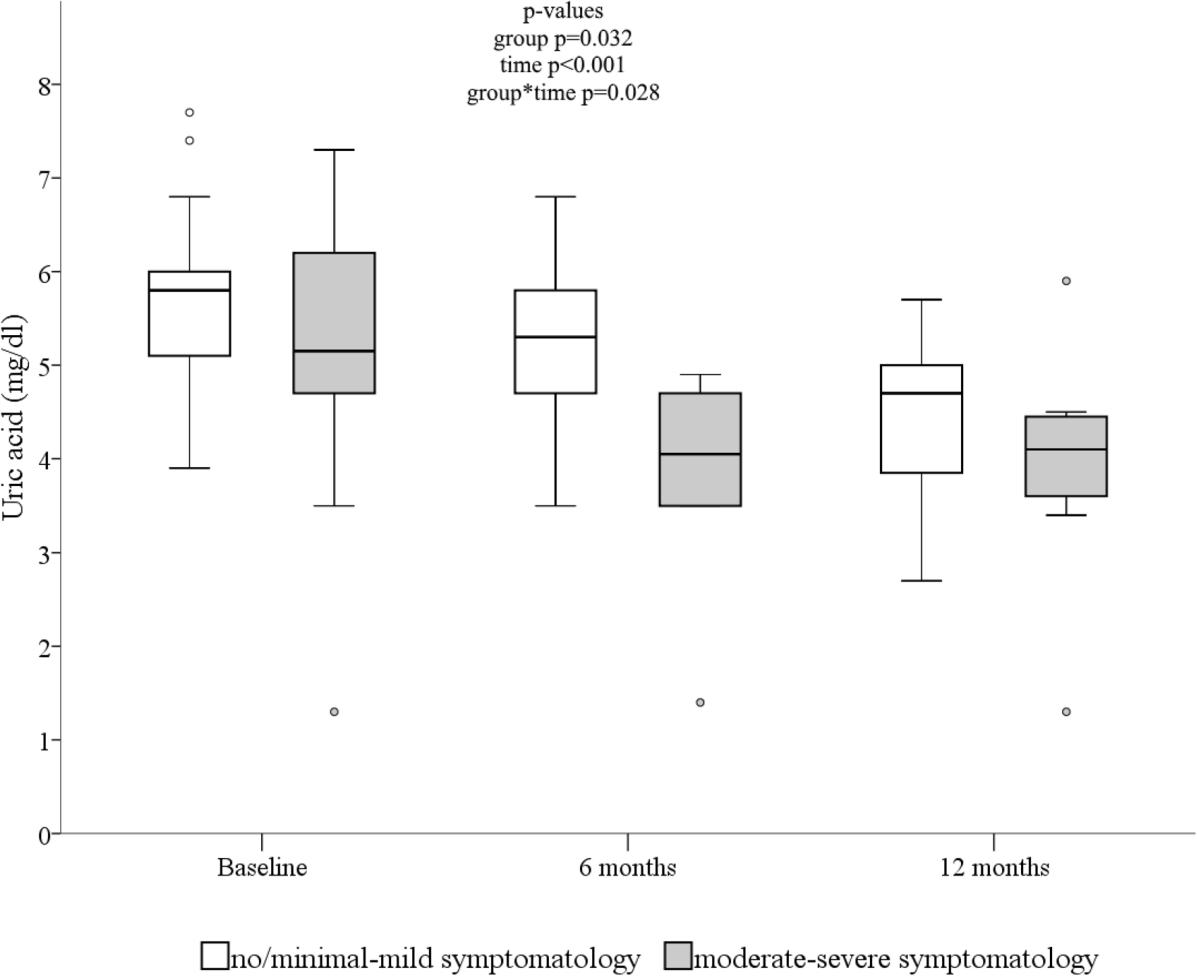
Change in serum uric acid according to depressive symptomatology over time. P values were based on Generalized Estimating Equations to examine the effects of time (from baseline to 6 and 12 months), group (depressive symptomatology), and their interaction on sUA as the dependent variable. Linear link function and an unstructured correlation matrix were used. Significance tests were performed with Wald χ^2^ (α = 0.05). The models were adjusted for age, sex, and season. Baseline data and the none/minimal to mild depressive symptomatology group were the reference.

Patients undergoing OAGB with moderate to severe depressive symptomatology presented with lower uric acid levels than patients with no or minimal to mild depressive symptomatology over time (Fig. 2).

ROC analysis revealed that sUA levels had a moderate to good prognostic accuracy in the total sample (at baseline: AUC 0.57, 95% CI 0.40–0.73; at 6 months: AUC 0.69, 95% CI 0.49–0.85; at 12 months: AUC 0.57, 95% CI 0.37–0.76).

The optimal cut-off levels for predicting moderate to severe depressive symptomatology in patients undergoing OAGB were 5.0 mg/dl (PPV of 71%; sensitivity: 46%; specificity: 77%) at baseline, 5.2 mg/dl (PPV of 71%; sensitivity: 88%; specificity: 53%) at 6 months, and 4.5 mg/dl (PPV of 71%; sensitivity: 78%; specificity: 50%) at 12 months (calculated by the Youden Index method).

### sUA levels and weight change

To investigate whether changes in sUA concentrations (delta sUA) were associated with anthropometric parameters, changes of weight, BMI and waist circumference to delta sUA levels were correlated. The changes were calculated as 12 months follow-up minus baseline. Furthermore, at 12 months, delta sUA was significantly correlated with delta weight (β = 0.379; p = 0.003), delta BMI (β = 0.473; p = 0.012), and delta waist circumference (β = 0.531; p = 0.003).

## Discussion

In this secondary analysis of a randomized trial with patients undergoing OAGB surgery, a high rate of depressive symptoms at T0/baseline was observed. There was an improvement in BDI total score and the subdivision scores at 6 and also at 12 months, except for the cognitive-affective subdivision at 12 months. BMI loss showed no association with depressive symptomatology at 6 and 12 months. Further, patients undergoing OAGB in both depressive symptomatology groups showed the same amount of weight at baseline. Moreover, sUA levels decreased significantly following bariatric surgery. Patients with moderate to severe depressive symptomatology presented with lower sUA levels than patients with no or minimal to mild depressive symptomatology. ROC analysis revealed that sUA levels of 5.0 or 4.5 mg/dl (T6 or T12) had a prognostic accuracy for depression severity in patients undergoing OAGB. Decrease in sUA correlated with changes in weight, BMI and waist circumference.

There is evidence, that bariatric surgery is a powerful tool for understanding the complex cascade of events associated with the regulation of body weight, improvement of health conditions, and mental health^32–35^. A prospective, well-controlled study, the Swedish Obese Subjects (SOS) study, involving 4047 patients with obesity assessed the change in psychological aspects postoperatively. This study demonstrated that depressive symptomatology decreased after weight reduction, but the decrease only remained stable among those who lost ≥ 25% of their initial weight^36^. Additionally, numerous studies, also using the BDI score, showed elevated depressive symptomatology in patients undergoing bariatric surgery^25,37–44^. The results from the present study are also comparable to the results of a study by Mitchell et al.^25^, where the authors reported on a significant decline in reported depression scores among patients within the first 6 months, after which the scores remained steady at the 1 year follow up. Interestingly, after the 1 year mark there was an observed modest, yet significant increase in the depression scores at 2 and 3 year follow up^25^. It is hypothesized that this deterioration in mood improvement may be linked to unrealistic expectations of the surgical outcomes and the related disappointment^45^, weight regain and/or reoccurrence of comorbiditie^46,47^, various nutritional deficiencies, which theoretically could present with depressive features^48^ or relative malabsorption of antidepressants^49,50^.

The results from the present study are consistent with previous literature^28,29^, where patients undergoing bariatric surgery are more likely to endorse more somatic and vegetative symptoms, rather than mental aspect of depression. As with the BDI it is possible to distinguish between cognitive-affective and somatic-vegetative items by using subscales. With these subscales it might be possible to evaluate whether the symptoms are depressive ones or concomitant symptoms of obesity, which may be important for a clinician to see if a high BDI total score is being primarily driven by concomitant symptoms of obesity^29^.

Moreover, some authors have found that patients with depressive symptomatology presented poor weight or BMI loss after surgery^38,51–53^. One explanation was that the former affects one’s ability to adapt to post-surgical behavioural changes^53^. However, the results from the present study showed that the improvement in BDI score was independent of the magnitude of BMI loss. These are in line with a study from Ayloo et al.^37^, which observed that weight loss had no independent influence on BDI improvement. The results indicated that the change in mood following bariatric surgery is related to the multiple factor interaction including initial weight loss. Here, psychosocial factors possibly have a more substantial role in the change in mood than was previously known^37^.

To our knowledge, this is the first analysis that compares levels of sUA and depressive symptomatology in patients undergoing OAGB. sUA levels demonstrated a moderate to good prognostic accuracy as a biomarker for patients with depressive symptomatology and sUA levels were higher with moderate to severe depressive symptomatology.

Another possible mechanism may be related to the effects of depression in decreasing antioxidant defences and increasing oxidative stress as reported^54,55^. The few studies that, to date, investigated the association between plasma uric acid levels and depression report conflicting results. These were mostly case–control studies that found increased^56,57^, decreased^58,59^, and no difference^60^ in levels of uric acid. Recently published meta-analyses has shown that participants with major depressive disorder have levels of the antioxidant uric acid lower than healthy controls^12,61,62^. The results could have broader implications given previous hypotheses regarding oxidative stress status^63^ and purinergic role^64^ in major depressive disorder.

Another possible explanation of the association between depression and uric acid could be the dietary habits of patients with obesity (e.g., increased sugar-sweetened soft drinks intake, fructose consumption, and increased purines intake)^65^, as lifestyle factors, including alcohol use, decreased physical activity and smoking. All these factors are associated with oxidative stress and are found to be more prevalent in people with depression. Increased oxidative stress could lead to depletion of antioxidants, including uric acid. A longitudinal cohort study included these factors and determined little effect on the association in major depressive and anxiety disorders^66^.

Moreover, a pilot study investigated the association between sUA concentrations and loss of body weight following laparoscopic sleeve gastrectomy or laparoscopic Roux-en-Y-gastric bypass in severely adolescents with morbid obesity^67^ and demonstrated a strong association of sUA change with changes in body weight. The authors hypothesized that sUA concentration may be influenced by factors that bariatric surgery procedures have in common, namely change in weight, adiposity, and dietary patterns.

This secondary analysis has certain limitations. First, the sample size is rather small, although it was based on the sample size calculation taking into account differences of serum 25(OH)D concentrations at 6 months between the intervention and control group. Indeed, we were able to demonstrate significant differences, which validate the sample size calculation. This study included a high percentage of women (80%), which, however, is very common in candidates for bariatric surgery, and might thus not be generalized to a male population. Another limitation might be that the parent study is a randomized trial of vitamin D supplementation. Evidence shows an association between vitamin D and uric acid levels^68^ and between vitamin D and depression^69–71^. However in this secondary analysis there was no association between vitamin D and uric acid levels and between vitamin D and depression, therefore vitamin D is not a confounding factor on this associations.

The strengths of the study include a detailed pre- and postoperative data of patients undergoing OAGB, which, as a rather new bariatric procedure, has not been evaluated in that regard. Furthermore, this study showed that sUA levels are associated with depressive symptomatology in patients undergoing OAGB.

## Conclusion

In conclusion, this study reports in patients undergoing the relatively new OAGB an improvement in depressive symptomatology postoperatively after 6 and 12 months, which was not associated with BMI loss. Furthermore, sUA levels also decreased significantly postoperatively. Patients with moderate to severe depressive symptomatology presented lower sUA levels than patients with none or minimal to mild depressive symptomatology. This decrease correlated with changes in weight, BMI and waist circumference. sUA levels below 5 mg/dl had a prognostic accuracy for depression severity in patients undergoing OAGB, which might be useful in clinical practice.
